# How youth engagement can break surgery out of its silo in global health

**DOI:** 10.5588/pha.23.0027

**Published:** 2023-09-21

**Authors:** R. Dutta, H. Sana, R. Sawhney, O. El Omrani, A. Ehsan, P. Fallah, M. Pigeolet, A. Jayaram, R. Riviello, K. B. Park

**Affiliations:** 1 Program in Global Surgery and Social Change, Department of Global Health and Social Medicine, Harvard Medical School, Boston, MA,; 2 Department of Obstetrics and Gynecology, Massachusetts General Hospital, Boston, MA, USA;; 3 WHO Collaborating Centre for Research in Surgical Care Delivery in Low and Middle-Income Countries, Mumbai, India;; 4 Plastic and Reconstructive Surgery Department, Ain Shams University Hospital, Cairo, Egypt;; 5 Department of Surgery, Brigham and Women’s Hospital, Boston, MA,; 6 Department of Obstetrics and Gynecology, Brigham and Women’s Hospital, Boston, MA, USA;; 7 Department of Pediatric Orthopedics, Húpital universitaire Necker - Enfants malades, Paris, France;; 8 Department of Surgery, Beth Israel Deaconess Medical Center, Boston, MA, USA;; 9 Center for Equity in Global Surgery, University of Global Health Equity, Kigali, Rwanda

In 2015, the Lancet Commission on Global Surgery reported that approximately 18 million deaths occur every year due to inadequate access to surgical care.^1^ Concurrently, the 194 WHO Member States unanimously adopted World Health Assembly (WHA) resolution 68.15, acknowledging the critical role of surgery in achieving universal health coverage (UHC).^2^ Despite this, the prioritization of surgery within the broader WHO agenda has been insufficient, with the issue being overlooked in the framing of the Sustainable Development Goals (SDGs) in 2016.^3^

Youth-led organizations such as the International Students Surgical Network have been instrumental in advocating for global surgery, using social media campaigns, educational resources, and attendance at high-level meetings to push their agenda. The youth retain innovative thinking competencies with untapped potential and a unique ability to become vectors for change. The discoveries of heparin, insulin, the sinoatrial node and ether anesthesia are all attributed to medical students. This is testament to the impact that can be created in healthcare if young people receive opportunities.

The WHA side events are an avenue for bringing together stakeholders; however, despite being a key stakeholder in the current and future of the surgical workforce, global youth engagement in surgery is lagging in this sphere. Youth voices remained underrepresented in the 75^th^ WHA in 2022, which had only 13 surgical representatives out of almost 6,000 delegates present, none of whom were from the youth demographic, and which had limited influence.^4^ To break the current silo and integrate the perspectives of young global surgery advocates, the WHO needs to establish a mechanism by providing dedicated quotas to ensure youth presence.

Established in 2021, the WHO Youth Council engages the youth in setting the course of action and influencing priorities with the Director General and senior leadership of the WHO. The Steering Committee comprises 22 youth organizations from health and non-health sectors, representing global health and noncommunicable diseases (NCD) coalitions, but not the surgical community.^5^ There are 1.8 billion people between the ages of 10 and 24 – the largest youth population to ever exist in history – and their needs must be addressed to achieve the overarching SDG 2030 agenda.^6^ It is essential to meaningfully engage global surgery youth in decision-making tables at the highest levels of WHO and civil society initiatives, especially as the WHO prepares to release the first progress report on resolution 68.15 and global surgical civil societies establish advocacy strategies. One crucial step is to provide dedicated spots and adequate floor time for global surgery youth in WHO meetings, as well as to invite global surgery youth organizations to join the WHO Youth Council to address the youth burden of surgical diseases.^7^

Furthermore, the global surgery civil society must actively engage youth advocates in all policy and advocacy efforts through side events, meetings, and toolkits, both during and beyond the WHA.^8^ As the global health community convenes at the 77^th^ WHA in 2024 to discuss NCDs towards achieving UHC 2030, it is imperative to include youth voices to weave surgery into the global health and NCD discourse to impact the health, future, and prosperity of youth for generations to come.
